# Exogenous Melatonin Modulates Photosynthesis and Antioxidant Systems for Improving Drought Tolerance of Sorghum Seedling

**DOI:** 10.3390/cimb46090581

**Published:** 2024-09-03

**Authors:** Yushan Bo, Yifan Xing, Yu Wang, Wendong Gu, Xinyi Jiang, Jiarui Yu, Xiaolong Shi, Chunjuan Liu, Chang Liu, Yufei Zhou

**Affiliations:** College of Agronomy, Shenyang Agricultural University, Shenyang 110866, China; 15998258250@163.com (Y.B.); yfxing1996@163.com (Y.X.); ws2474456845@gmail.com (Y.W.); 15737663326@163.com (W.G.); c1135911553@163.com (X.J.); yujiar2018@163.com (J.Y.); xiaolongshi@syau.edu.cn (X.S.); liuchunjuan@syau.edu.cn (C.L.); liuchang@syau.edu.cn (C.L.)

**Keywords:** sorghum, melatonin, antioxidant enzymes, non-enzymatic antioxidants, photosynthesis, water deficit

## Abstract

Sorghum faces significant production challenges due to drought stress. Melatonin has been demonstrated to play a crucial role in coping with stresses in plants. This study investigated the effect of exogenous melatonin on the sorghum seedling growth, photosynthetic capacity, and antioxidant system under drought stress. The results indicated that drought stress inhibited the growth of sorghum seedlings by a marked reduction in leaf relative water content, along with a significant increase in both malondialdehyde and hydrogen peroxide content. The drought stress also led to a significant diminution in chlorophyll contents, thereby curtailing the capacity for light energy capture. Furthermore, the efficiency of the photosynthetic electron transport chain was adversely impacted. However, the application of exogenous melatonin notably mitigated the adverse effects on sorghum seedlings under the drought stress. Additionally, it stimulated an elevation in the photosynthetic rate and a decrease in non-photochemical quenching. The exogenous melatonin also facilitated the preservation of the chloroplast ultra-structure and boosted the activity of antioxidant enzymes and the content of non-enzymatic antioxidants. Cluster heat maps and principal component analysis further revealed significant correlations among various parameters under different treatment conditions. These results highlight melatonin’s role in improving sorghum’s drought tolerance, which is beneficial for agricultural management.

## 1. Introduction

In the face of global climate change and the increasing conflict between water supply and demand, drought has emerged as a prevalent issue within agricultural production. The repercussions of this phenomenon on crop yields, quality, and profitability are anticipated to escalate in the coming years [1]. Consequently, enhancing the drought tolerance of crops is imperative for maintaining robust and consistent grain production. *Sorghum bicolor* (L.) Moench, a vital crop for both food security and as a source of renewable energy, is extensively cultivated across the world, particularly in regions characterized by arid and semi-arid climates [2]. While sorghum is inherently resilient to arid conditions, its frequent exposure to such environments predisposes it to seasonal and intermittent droughts. These are especially impactful during the critical germination and early growth stages. Particularly during these early seedling stages of development, drought has emerged as the most substantial abiotic stressor, significantly hindering the growth of sorghum [3]. Therefore, it is crucial to understand the physiological response of sorghum to drought stress and develop suitable production management strategy.

Under drought conditions, plants produce a large number of reactive oxygen species (ROS), which can oxidize biological macromolecules in cells, hinder normal plant metabolism and growth, and even lead to death [4]. To cope with this oxidative stress, plants have developed a complex antioxidant system to eliminate excess ROS and maintain the redox balance within cells [5]. The antioxidant system mainly includes enzymatic and non-enzymatic antioxidant systems. The enzymatic antioxidant system involves various enzymes, such as superoxide dismutase (SOD), peroxidase (POD), catalase (CAT), and ascorbate peroxidase (APX), which work in concert to neutralize ROS and maintain cellular redox homeostasis. For instance, SOD catalyzes the dismutation of superoxide radicals into oxygen and hydrogen peroxide, which is then further detoxified by CAT and APX [6]. The non-enzymatic antioxidant system includes a range of small molecule antioxidants, such as ascorbic acid (AsA), glutathione (GSH), flavonoids, and carotenoids, which can directly scavenge ROS or participate in antioxidant reactions as auxiliary factors. The ascorbate–glutathione cycle is particularly important because it helps regenerate AsA and GSH, both of which are directly involved in ROS detoxification [7]. Under drought stress, the plant’s antioxidant system undergoes changes to adapt to environmental pressures. For example, some studies have shown that drought stress leads to an increase in the activity of antioxidant enzymes in plants, enhancing their ability to eliminate ROS [8]. Sher et al. [9] showed that sorghum alleviates drought stress by adjusting its antioxidant defense system. However, the response of the antioxidant system may vary among different plant species and under different degrees of drought stress [10]. Hence, there is a great interest in understanding how the antioxidant system plays a role in maintaining the normal physiological functions of plants.

Drought stress is well documented to impact photosynthesis through various physiological mechanisms, leading to reduced carbon assimilation and altered electron transport rates [11]. In addition to the well-known effects, it is important to note that several plant hormones, including abscisic acid, cytokinins, gibberellins, and brassinosteroids, play crucial roles in modulating plant responses to water deficit conditions. These hormones coordinate a complex signaling network that helps plants to adapt and acclimate to drought stress [12,13]. Melatonin, as a hormone-like compound, has been shown to exert a positive influence on these responses.

Melatonin, a tryptophan-derived natural product, plays a pivotal role in plant growth and stress responses. It is naturally produced in plants, with synthesis occurring in various organs such as leaves, roots, and seeds, particularly under conditions of environmental stress [14]. The exogenous application of melatonin has shown promise in enhancing stress tolerance in crops, where it ameliorates the negative impacts of adverse conditions and improves overall plant performance. For instance, Guo et al. [15] discovered that exogenous melatonin mitigates the damage to the photosynthetic apparatus of corn seedlings under drought stress by enhancing the efficiency of photosynthetic electron transport and the photochemical quantum yield of PSI and PSII. Wang et al. [16] demonstrated that exogenous melatonin strengthens photosynthesis in tomatoes under cold stress by increasing the content of photosynthetic pigments and the activity of photosystem II. Jahan et al. [17] showed that melatonin alleviates the toxicity of nickel to tomato seedlings by improving photosynthesis and enhancing tolerance to secondary metabolism and oxidative stress. These studies indicate that under stress conditions, melatonin improves photosynthetic efficiency, helping to maintain the stability of photosynthetic products [18,19]. Moreover, melatonin can also maintain the integrity of photosynthetic organs under environmental stress. Stress-induced melatonin synthesis may be a self-protection strategy for plants, where melatonin synthesis in chloroplasts can protect the photosystems from oxidative damage [19]. Additionally, melatonin can also increase the activity of plant antioxidant enzymes, enhancing the plant’s resistance to abiotic stress [20,21]. For example, Arnao and Hernández-Ruiz [22] reviewed the multiple functions of melatonin in plants, including its role in the antioxidant system. Most of the key enzymes required for melatonin synthesis are located in the chloroplasts, suggesting that melatonin may play an important role in the function of chloroplasts [23,24].

Although previous studies have indicated the potential of melatonin in enhancing the drought tolerance of plants [25,26], the specific regulatory mechanisms of its effects on photosynthesis and antioxidant metabolism in sorghum seedlings are not yet clear. This study aims to explore how exogenous melatonin can improve the adaptability of sorghum seedlings under drought stress by regulating their photosynthesis, chlorophyll fluorescence, and electron transport processes, and to reveal the mechanism by which melatonin regulates the antioxidant system.

## 2. Materials and Methods

### 2.1. Plant Material

This study was conducted in the sorghum physiology laboratory of Shenyang Agricultural University. We utilized the sorghum variety ‘jiza138’ as our experimental material. Uniform sorghum seeds were selected and disinfected with a 5% sodium hypochlorite (NaClO) solution for 10 min. After being rinsed with distilled water, the seeds were placed into petri dishes with moistened filter paper and incubated in a laboratory incubator (AR-41L3 Flex, Pervical, Hong Kong) under the following conditions: a temperature at 28/25 °C day/night and a photoperiod of 12 h of light followed by 12 h of darkness. After 2 days, well-developed seedlings were selected and transferred into hydroponic boxes filled Hoagland’s nutrient solution, with 16 plants per box. The nutrient solution was changed every 3 days, and the seedlings were cultivated until they reached the two-leaf stage, at which point the treatment commenced.

### 2.2. Experimental Design

This experiment encompassed four distinct treatments. Treatment 1: CK (Control, with normal water supply); Treatment 2: D (drought, 20%PEG); Treatment 3: D + MT (application of 100 μmol/L melatonin under drought stress); Treatment 4: D + P (application of 100 μmol/L melatonin inhibitor under drought stress). When the sorghum seedlings reached the two-leaf stage, treatment 3 and treatment 4 were sprayed with their respective concentrations of melatonin and melatonin inhibitor for 3 days continuously, ensuring that the spray was evenly distributed without dripping. Upon completion of the exogenous melatonin or melatonin inhibitor spraying, the drought stress was simulated using PEG-6000 at a concentration of 20%. Treatments 3 and 4 were sprayed with melatonin and melatonin inhibitor, respectively, prior to the drought treatment to assess the prophylactic potential of melatonin in inducing stress resistance in sorghum seedlings. The timing of the application was chosen to coincide with a critical growth phase known for heightened sensitivity to external stimuli, potentially allowing for a more pronounced activation of the plant’s stress-response pathways [26]. Each treatment was replicated at least four times. After 7 days of exogenous application of melatonin or melatonin inhibitors, uniform sorghum seedlings were selected; the plant height (PH), root length (RL), shoot fresh weight (SFW), root fresh weight (RFW), shoot dry weight (SDW), and root dry weight (RDW) were measured; and the first fully expanded leaf from the top was collected for further analysis.

### 2.3. Relative Leaf Water Content

The relative water content of the first fully expanded leaf from the top was determined using the saturated weighing method [27]. Fresh sorghum leaves were selected, weighed for fresh weight (FW), soaked in distilled water overnight, and then surface-dried. The saturated weight with absorbed water (TW; turgid weight) was recorded, followed by drying in an oven at 105 °C for 10 min and then at 50 °C until a constant weight was achieved for the dry weight (DW). Each treatment was replicated four times. RWC was calculated as follows:

RWC (%)=[(FW−DW)/TW−DW]×100

### 2.4. Leaf Pigment Content and Photosynthetic Parameters

#### 2.4.1. Determination of Leaf Pigment Content

A hole punch with a diameter of 1.2 mm was used to sample the first fully expanded leaf from the top, which was then placed in a dark reagent bottle containing 10 mL of 80% acetone. Total chlorophyll was extracted after 24 h of dark storage. The contents of chlorophyll a (Chla), chlorophyll b (Chlb), total chlorophyll (Chl), and carotenoid (Car) were determined using a spectrophotometer (UV-2550, Shimadzu, Tokyo, Japan). Calculation formulas are as follows:

Chlorophyll a concentration (mg L^−1^): Chla = 13.95A665 − 6.8A649

Chlorophyll b concentration (mg L^−1^): Chlb = 24.96A649 − 7.32A665

Carotenoid concentration (mg L^−1^): Car = (1000A470-2.05Ca − 114.8Cb)/248

Total chlorophyll content (mg g^−1^ FW) = chlorophyll a content + chlorophyll b content

where A665, A649, and A470 are the optical densities of chlorophyll solutions at wavelengths of 665, 649, and 470 nm, respectively [28].

#### 2.4.2. Determination of Leaf Photosynthetic Parameters

The net photosynthetic rate (Pn), stomatal conductivity (Gs), transpiration rate (Tr), and intercellular CO_2_ concentration (Ci) were measured using a Li-6400 photosynthetic apparatus (LI-COR Corporation, Los Angeles, CA, USA). The measurements were taken from the first fully expanded leaf from the top of the sorghum seedling, with each treatment repeated three times. The parameters were set to light intensity of 1000 μmol·m^−2^·s^−1^, a CO_2_ concentration of 385 ± 5 μmol·mol^−1^, while the temperature was 28 °C. The measured leaf compartment area was 2 cm^2^.

### 2.5. Chlorophyll Fluorescence Parameters, Relative Electron Transport Efficiency Light Response Curve, and OJIP Measurement

#### 2.5.1. Determination of Chlorophyll Fluorescence Parameters

The first fully expanded leaf from the top of the sorghum seedlings was selected for measurement, with four replicates per treatment. After dark adaption at room temperature for 30 min, the leaves were measured using a FlourCam FC 800-O/2020 chlorophyll fluorometer (Brno, Czech Republic). The measurement parameters were set as follows: the leaves were illuminated with a measurement light of less than 0.1 μmol·m·s^−1^ to determine the initial fluorescence (F0), followed by the application of a saturating pulse light (10,000 μmol·m^−2^·s^−1^, 0.7 s) to measure the maximum fluorescence (Fm). After 15 min of light adaptation (800 μmol·m^−2^·s^−1^), the maximum fluorescence (Fm′) was obtained. The main parameters obtained include Fv/Fm (the maximum photochemical efficiency of Photosystem II) and (Fm−Fm′)/Fm′ (the non-photochemical quenching, NPQ, which represents thermal dissipation).

#### 2.5.2. Relative Electron Transport Efficiency Light Response Curve and OJIP Determination

Utilizing the dual-channel fluorescence measurement system Dual-PAM-100 (WALZ, Bayern, Germany), photosynthetically active radiation (PAR) intensities were set at 19, 37, 47, 104, 184, 246, 416, 650, 1009, and 1548 μmol m^−2^ s^−1^. For each treatment, the electron transport rate (ETR) of the first leaf from the top of sorghum seedlings was randomly measured, with four repetitions, and the PAR-ETR response curves were plotted. The rapid light response curve parameters of ETR were fitted using the Platt rapid light response model, with the formula as follows:

$$ETR=Pm\times (1-e^{-\alpha \times PAR⁄Pm})\times e^{-\beta \times PAR⁄Pm}$$

where Pm is the maximum potential relative electron transport rate in the absence of light inhibition; α is the initial slope of the rapid light curve, indicating the efficiency of photon utilization; and β is the light inhibition parameter.

The Dual-PAM-100 was also used to measure the leaf OJIP curves (measured after 30 min of dark adaptation of the leaves). For each treatment, the first fully expanded leaf from the top was randomly measured, with four repetitions, and the OJIP curves were plotted.

### 2.6. Observation of Chloroplast Ultrastructure and Determination of Chloroplast Antioxidant Substances

#### 2.6.1. Observation of Chloroplast Ultrastructure

Using the first fully unfolded leaf from the top, the ultrastructure of chloroplasts was measured. Prior to sampling, the leaves were meticulously cleaned with distilled water, and any surface moisture of the leaves was removed using filter paper. Subsequently, small strips (1 mm × 3 mm) were excised avoiding the veins and preserved in a solution of 2.5% pentanediol. Samples were processed according to Shu et al. [29]. The samples were sectioned into 50 nm ultrathin slices using a Leica EM UC7 ultrathin microtome (Wuhan Servicevio Technology Co., Ltd., Wuhan, China). These slices were stained using uranium acetate and alcohol. Finally, the stained samples were observed with a transmission electron microscope.

#### 2.6.2. Determination of Antioxidants in Chloroplast

Chloroplasts were extracted by the non-enzymatic method. A 1 g sample of the first fully expanded fresh leaf from the top was selected. Then, 2 mL of phosphate-buffered saline (PBS) was added and the mixture was homogenized thoroughly using a blender. The homogenate was then filtered through a 100-micrometer cell sieve. The filtrate was centrifuged at 3000× *g* for 10 min twice, with the supernatant being discarded and the pellet collected each time. After centrifugation at 200× *g* and 1000× *g* for 2 min, the supernatant was collected and then centrifuged again at 3000× *g* for 10 min to collect the pellet, which contained the chloroplasts.

For the determination of antioxidants in chloroplasts, glutathione peroxidase (GPX) activity was measured according to the method by Nakano et al. [30]. Glutathione reductase (GR) activity was measured according to the method of Li et al. [31]. Monodehydroascorbate reductase (MDHAR) activity and dehydroascorbate reductase (DHAR) activity were measured according to the method of Murshed et al. [32]. The content of reduced ascorbic acid (ASA) and dehydrogenase (DHA) was determined using the dipyridyl method, referring to the method of Aravind et al. [33]. The reduced glutathione (GSH) and oxidized glutathione (GSSG) contents were measured using the DTNB cycle detection method, referring to the method of Griffith et al. [34]. The assay kits for these determinations were provided by Suzhou Greats Biotech Co., Ltd., Suzhou, China.

### 2.7. DAB Staining and Relative Conductivity Measurement

#### 2.7.1. DAB Staining of Leaves

A 6–8 cm section from the middle to upper part of the first fully expanded leaf from the top was taken for DAB staining, with four replicates per treatment. The leaf sections were immersed in DAB staining solution and kept in the dark at room temperature for 2 to 6 h. Subsequently, the leaf sections were soaked in 95% ethanol and treated at 40 °C for 3 to 16 h to facilitate the decolorization process. After the decolorization, the leaf sections were rinsed and then the excess water was carefully blotted away.

#### 2.7.2. Determination of Relative Leaf Conductivity

The determination of the electrolyte exudation rate was slightly modified according to the method of Hnilikova et al. (2019) [35]. For the first fully expanded leaf of leaf from the top, each treated sample was washed several times with distilled water to remove the surface electrolyte, the tissue was immersed in distilled water at room temperature (25 °C) for 24 h, and the initial conductivity of the solution (R1) was measured using a conductivity meter (DDSJ-308F, Shjingmi, Shanghai, China). Finally, the sample was released in a boiling bath for 20 min, and cooled to room temperature to determine the final conductivity (R2). The relative conductivity (RC) was calculated as follows:


RC (%)=R1⁄R2×100

#### 2.7.3. Determination of Hydrogen Peroxide Content

The content of hydrogen peroxide was determined according to the method of Velikova et al. (2000) [36]. First, 0.1 g of fresh tissue sample was ground in 5 mL of 0.1% trichloroacetic acid until it became a homogenate. Then, it was centrifuged at 12,000× *g* for 20 min in a 4 °C centrifuge. Next, 0.5 mL of supernatant was mixed with 0.5 mL of 10 mM phosphate buffer solution (pH 7.0) and 1 mL of 1 M potassium iodide. The mixture was left to stand in the dark for 1 h, and the absorbance was measured at a wavelength of 390 nm.

### 2.8. Antioxidant Enzyme Activity and MDA Content in Leaves

To determine the MDA content in the first fully expanded leaf from the top of sorghum seedlings, 1 g of sorghum leaf was chosen; extraction solution (10% trichloroacetic acid, reaction solution is 0.5% thiobarbituric acid) and a small amount of fine quartz sand was added, then ground; then 8 mL of extraction solution was added to a 10 mL centrifuge tube, centrifuged at 4000 r/min for 10 min, and the supernatant was taken as the reaction solution. Further determination was carried out according to the method of Xia et al. [37]. This antioxidant extract can be used to determine its activity [37]. SOD activity was measured using the methods of Beyer and Fridovich (1987) [38]. The POD activity was determined using the Fielding and Hall (1978) [39] method. The determination of CAT activity follows the method of Chen et al. (2022) [40].

### 2.9. Determination of Leaf Proline and Soluble Sugars

#### 2.9.1. Determination of Proline Content in Leaves

To determine the proline content, 0.1 g fresh tissue sample was ground into homogenate in a 3% sulfosalicylic acid solution, and then heated in a water bath at 100 °C for 10 min. After cooling, it was centrifuged at 10,000× *g* for 15 min in a 4 °C centrifuge. The proline content in the supernatant was detected using ninhydrin reagent, and the absorbance was measured at 520 nm [41].

#### 2.9.2. Determination of Soluble Sugars in Leaves

A 0.3 g sample of the first fully expanded leaf from a sorghum seedling was weighed and powdered before being mixed together with 5 mL of anhydrous ethanol and quartz sand, 1 mL of the soluble sugar extract, and 6 mL of anthrone reagent, and the mixture was cooled for 10 min before measurement at 625 nm [42].

### 2.10. Determination of Melatonin Content in Leaves

A 0.2 g sample of the first fully expanded leaf from the top of the sorghum seedlings was weighed and 0.9 mL of PBS (pH 7.4) was added. The sample was homogenized thoroughly in an ice bath, and after centrifugation at 4 °C at 3000 revolutions per minute (rpm) for 30 min, the supernatant was removed. The content of melatonin in the leaf was determined using an ELISA kit (Jiangsu Meimian Industrial Co., Ltd., Yancheng, China).

### 2.11. Statistical Analysis

The data from at least three replicates were presented as mean ± SD and were analyzed using SPSS software (version 22.0, IBM, Chicago, IL, USA). One-way analysis of variance was performed and the LSD test was used to compare the treatment differences at *p* ≤ 0.05. Figures were drawn using Origin 2021.

## 3. Results

### 3.1. Effects of Exogenous Melatonin on Growth and Water Status of Sorghum Seedlings under Drought Stress

#### 3.1.1. Plant Growth

**Figure 1:**
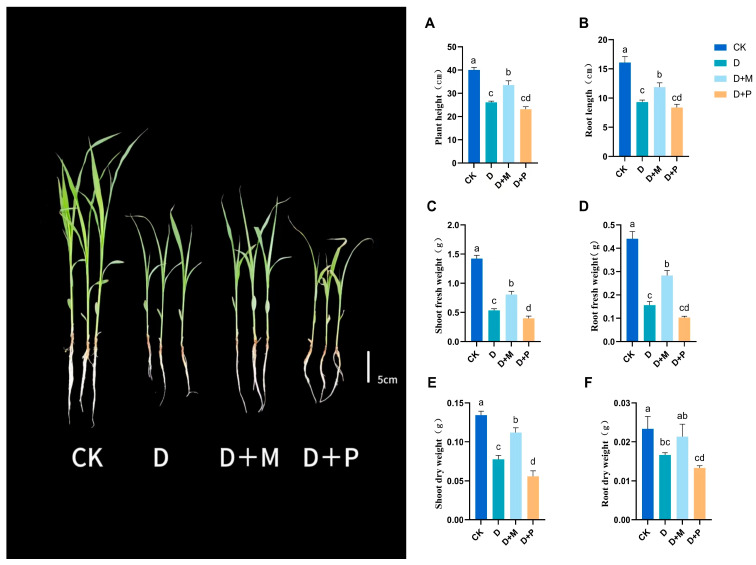
Exogenous melatonin promotes the growth of sorghum seedlings under drought stress conditions: (**A**) plant height; (**B**) root length; (**C**) shoot fresh weight; (**D**) root fresh weight; (**E**) shoot dry weight; (**F**) root dry weight. CK: control treatment; D: 20% PEG; D + M: 20% PEG + 100 μmol/L of MT; D + P: 20% PEG + 100 μmol/L of CPA; columns represent mean ± SD, and, according to the LSD test, *n* = 3, significant differences are indicated in lower case letters (*p* ≤ 0.05).

Drought stress significantly inhibited the growth of sorghum seedlings (Figure 1). Under drought stress, the plant height and root length of sorghum seedlings decreased by 34.86% and 42.24% compared to the control, respectively, and the fresh weight of the shoots and roots decreased significantly by 62.42% and 64.59%, respectively. Additionally, the dry weight of the shoots and roots significantly decreased by 42.1% and 28.57%, respectively. The application of exogenous melatonin mitigated the negative effects of drought stress, leading to a notable increase in growth. The plant height and root length increased significantly by 28.60% and 27.59%, respectively, and the fresh weight of the shoots and roots increased by 51.12% and 82.83%, respectively. The dry weight of the shoots significantly increased by 42.20%, and the dry weight of the roots increased by 28%. Conversely, the application of melatonin antagonist under drought stress inhibited the growth of sorghum seedlings, with the plant height and root length decreasing by 11.36% and 10.03%, respectively, and the fresh weight of the shoots and roots significantly decreasing by 29.24% and 39.97%, respectively. The dry weight of the shoots significantly decreased by 27.89%, and the dry weight of the roots decreased by 20%.

#### 3.1.2. Leaf Water Status

**Figure 2:**
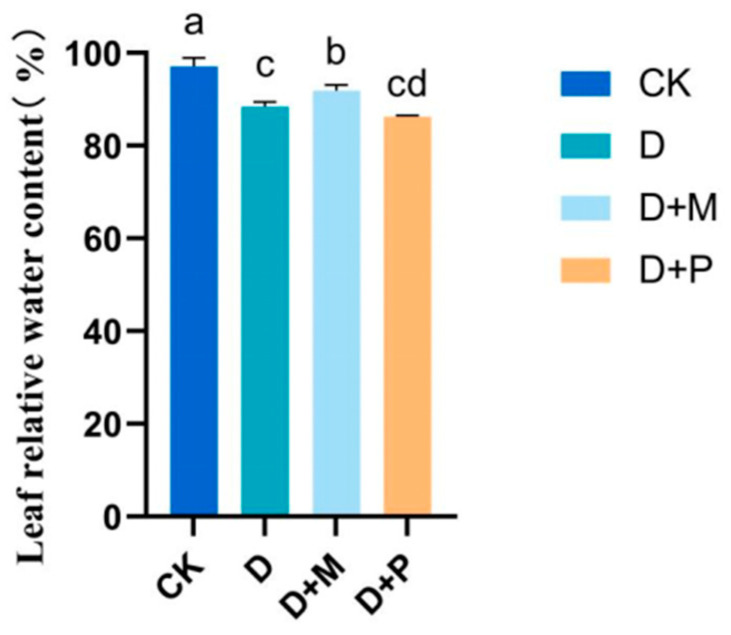
Effects of exogenous melatonin on relative water content of sorghum seedlings under drought stress. CK: control treatment; D: 20% PEG; D + M: 20% PEG + 100 μmol/L of MT; D + P: 20% PEG + 100 μmol/L of CPA; columns represent mean ± SD, and, according to the LSD test, *n* = 3, significant differences are indicated in lower case letters (*p* ≤ 0.05).

Drought stress notably reduced the RWC of sorghum leaves (Figure 2). Under drought stress, the RWC decreased by 10.66% compared to the control. The application of exogenous melatonin under drought stress improved the RWC of the sorghum leaves, with a significant increase of 7.07%. However, after the application of melatonin antagonist under drought stress, the RWC decreased by 2.46%.

### 3.2. Effects of Exogenous Melatonin on Photosynthetic Characteristics of Sorghum Seedlings under Drought Stress

#### 3.2.1. Effects of Exogenous Melatonin on Chlorophyll Content of Sorghum Seedlings under Drought Stress

**Figure 3:**
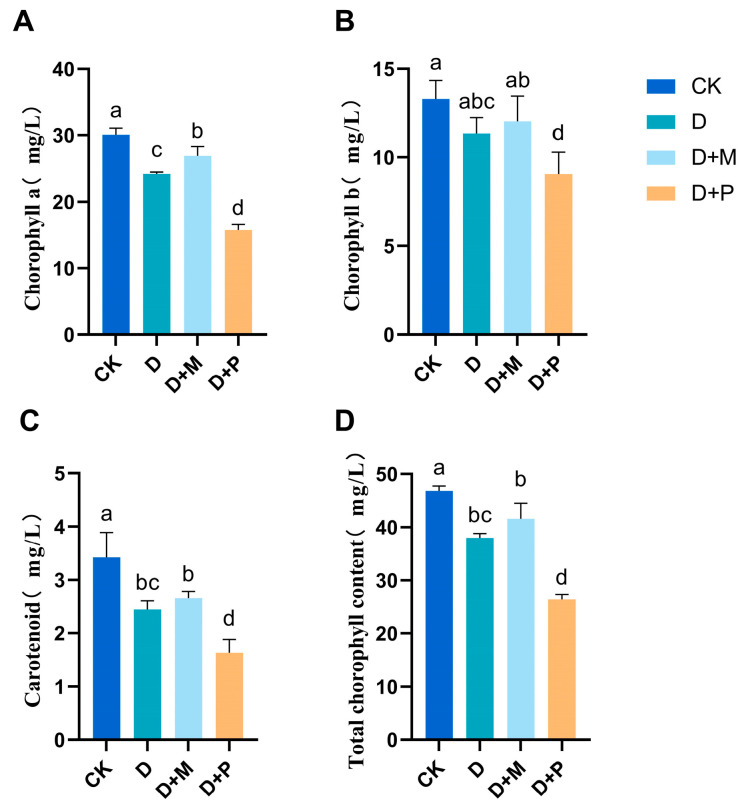
Effect of melatonin spraying on chlorophyll content in sorghum seedlings under drought stress: (**A**) chlorophyll A; (**B**) chlorophyll b; (**C**) carotene; (**D**) total chlorophyll. CK: control treatment; D: 20% PEG; D + M: 20% PEG + 100 μmol/L of MT; D + P: 20% PEG + 100 μmol/L of CPA; columns represent mean ± SD, and, according to the LSD test, *n* = 3, significant differences are indicated in lower case letters (*p* ≤ 0.05).

Under drought stress, the chlorophyll content of sorghum seedling leaves was markedly diminished (Figure 3). Compared to the control, the levels of chlorophyll a, chlorophyll b, carotenoids, and total chlorophyll under drought stress were significantly decreased by 19.50%, 14.73%, 28.50%, and 18.80%, respectively. In contrast to drought stress, the application of exogenous melatonin under drought stress increased the chlorophyll content in the sorghum seedlings, with significant increases of 11.04% for chlorophyll a and 8.74% for carotenoids, and observed increases in the levels of chlorophyll b and total chlorophyll. However, the application of melatonin antagonist under drought stress exacerbated the decrease in the chlorophyll content, with significant reductions of 35.04% for chlorophyll a, 33.35% for carotenoids, 30.46% for the total amount of chlorophyll, and 20.07% for chlorophyll b.

#### 3.2.2. Effects of Exogenous Melatonin on Leaf Photosynthetic Parameters of Sorghum Seedlings under Drought Stress

**Figure 4:**
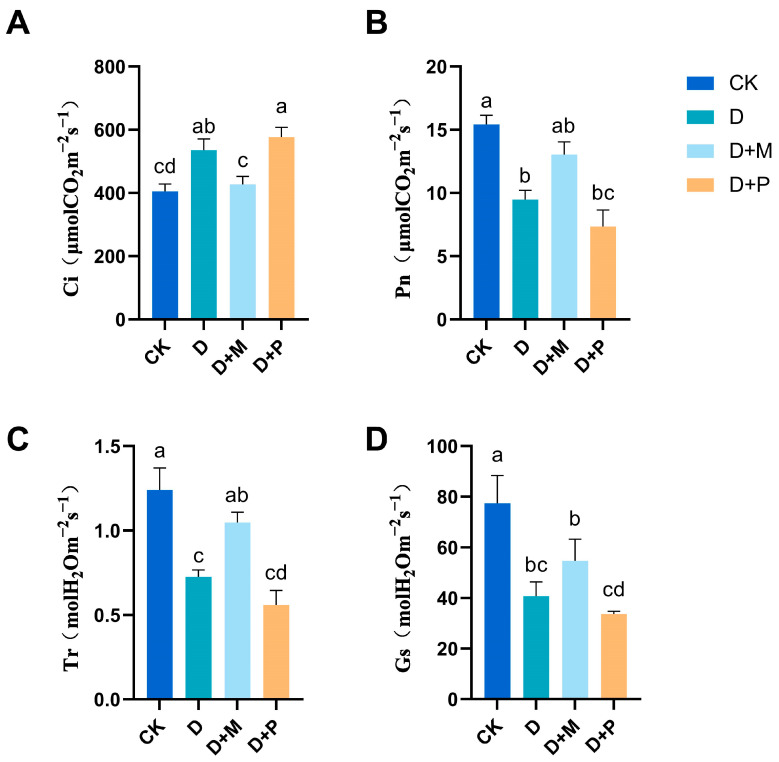
Under drought conditions, exogenous melatonin promotes the gas exchange of sorghum leaves: (**A**) Ci; (**B**) Pn; (**C**) Tr; (**D**) Gs. CK: control treatment; D: 20% PEG; D + M: 20% PEG + 100 μmol/L of MT; D + P: 20% PEG + 100 μmol/L of CPA; columns represent mean ± SD, and, according to the LSD test, *n* = 3, significant differences are indicated in lower case letters (*p* ≤ 0.05).

Drought stress significantly inhibited the photosynthesis of sorghum seedling leaves (Figure 4). Under drought stress, the transpiration rate (Tr), stomatal conductance (Gs), and net photosynthetic rate (Pn) of sorghum leaves significantly decreased by 41.40%, 47.41%, and 38.66% respectively, compared to the control, while the intercellular CO_2_ concentration (Ci) significantly increased by 32.28%. The application of exogenous melatonin under drought stress ameliorated these effects, leading to substantial improvements in the photosynthetic parameters. Specifically, Tr increased by 44.04% and Pn by 37.68%, while Ci significantly decreased by 20.23%. In contrast, the application of melatonin antagonist under drought stress further impaired the photosynthetic parameters of sorghum seedling leaves.

#### 3.2.3. Effects of Exogenous Melatonin on Leaf Fluorescence Parameters of Sorghum Seedlings under Drought Stress

**Figure 5:**
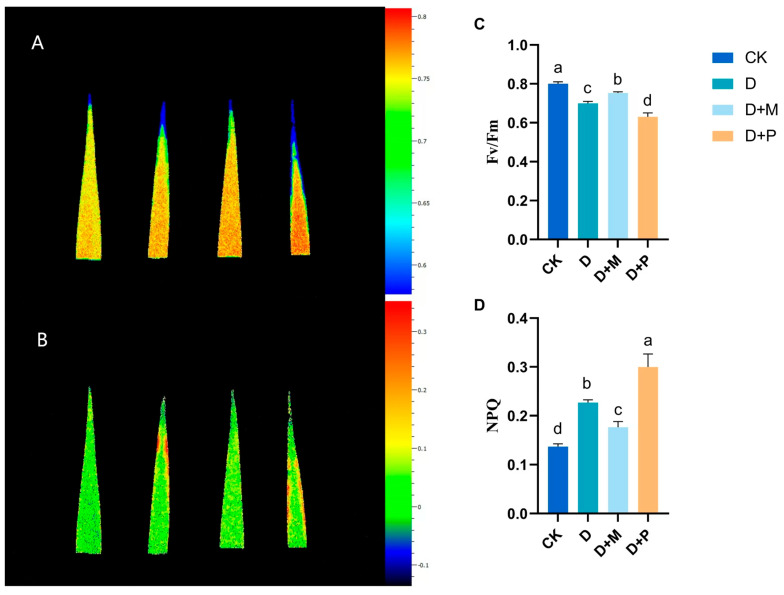
Under drought conditions, exogenous melatonin promoted the improvement of Fv/Fm and alleviated the increase in NPQ in sorghum leaves: (**A**,**C**) Fv/Fm; (**B**,**D**) NPQ. CK: control treatment; D: 20% PEG; D + M: 20% PEG + 100 μmol/L of MT; D + P: 20% PEG + 100 μmol/L of CPA; columns represent mean ± SD, and, according to the LSD test, *n* = 3, significant differences are indicated in lower case letters (*p* ≤ 0.05).

Drought stress profoundly affected the fluorescence parameters of sorghum seedling leaves (Figure 5). Compared to the control, the maximum photochemical efficiency of photosystem II (PSII), represented by Fv/Fm, significantly decreased by 12.5% under drought stress. However, the application of exogenous melatonin under drought stress alleviated this reduction, resulting in a significant increase of 7.61% in Fv/Fm. Conversely, the application of melatonin antagonist under drought stress exacerbated the reduction in Fv/Fm, leading to a significant decrease of 10%. Additionally, compared to the control, the non-photochemical quenching (NPQ) of sorghum seedling leaves significantly increased under drought stress, rising by 62.85%. The application of exogenous melatonin under drought stress mitigated this increase, with a significant decrease of 22.05% in NPQ. However, the application of melatonin antagonist under drought stress led to a similar increase in NPQ, with a significant rise of 32.35%.

#### 3.2.4. Effects of Exogenous Melatonin on Relative Electron Transfer Efficiency and OJIP

**Figure 6:**
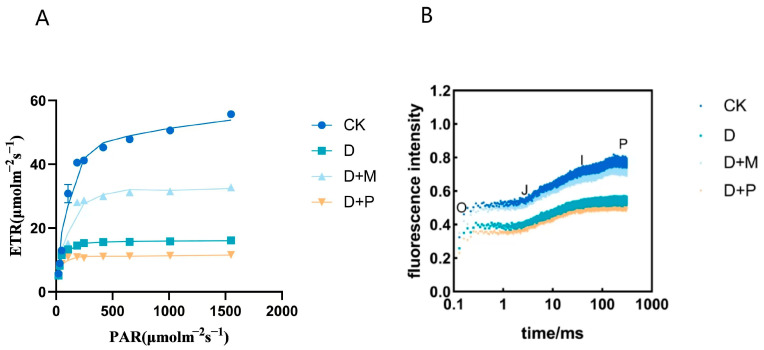
Under drought conditions, exogenous melatonin promoted the relative electron transport efficiency and fluorescence signal intensity in sorghum leaves: (**A**) Optical response curve of relative electron transfer efficiency; (**B**) OJIP. CK: control treatment. D: 20% PEG; D + M: 20% PEG + 100 μmol/L of MT; D + P: 20% PEG + 100 μmol/L of CPA.

The relative electron transport efficiency response curve is instrumental in interpreting the photosynthetic apparatus’s ability to capture light energy and transfer electrons. Compared to the control, the electron transport rate (ETR) exhibited a significant decline under drought stress (Figure 6A). After the application of exogenous melatonin, the ETR increased compared to drought treatment; however, the application of melatonin antagonist intensified the reduction in ETR compared to drought treatment.

Drought stress significantly diminished the fluorescence signal intensity in sorghum seedling leaves relative to the control (Figure 6B). The application of melatonin under drought stress resulted in an increase in fluorescence signal intensity in sorghum seedling leaves compared to drought treatment. Conversely, the application of melatonin antagonist under drought stress led to a decrease in fluorescence signal intensity compared to drought treatment. This indicated that drought stress inhibits the electron transport capacity on both the donor and acceptor sides of the photosystem II (PSII) reaction center, with a noticeable reduction in signal intensity in the treated leaves. In contrast, the application of exogenous melatonin preserved a robust OJIP signal, suggesting that it can mitigate the damage to the PSII reaction center inflicted by drought stress. This highlights the protective role of melatonin in maintaining the integrity of the photosynthetic apparatus under drought stress.

#### 3.2.5. Effects of Exogenous Melatonin on Chloroplast Ultrastructure of Leaves of Sorghum Seedlings under Drought Stress

**Figure 7:**
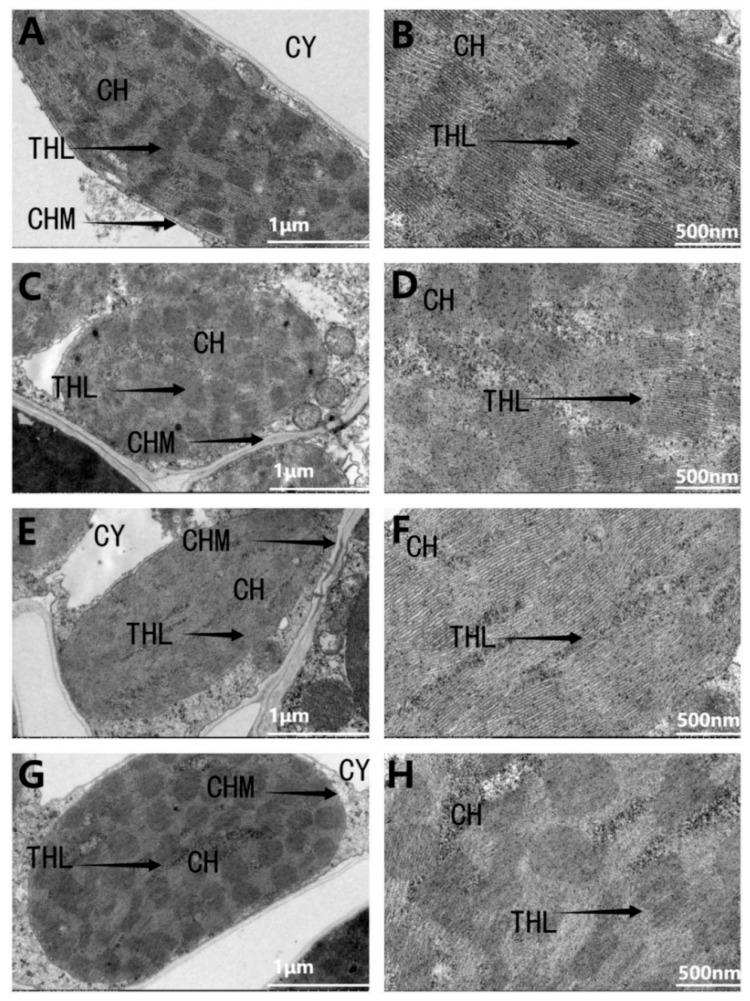
Effect of melatonin spraying on leaf chloroplast ultrastructure in sorghum seedlings under drought stress. CK: control treatment; D: 20% PEG; D + M: 20% PEG + 100 μmol/L of MT; D + P: 20% PEG + 100 μmol/L of CPA. CY-cytoplasm, CH-chloroplast, CHM-chloroplast membrane, THL-thylakoid lamella. Figure (**A**,**B**) are chloroplast 5000 and chloroplast 20,000 under CK treatment; (**C**,**D**) are chloroplast 5000 and chloroplast 20,000 under D treatment; (**E**,**F**) are chloroplast 5000 and chloroplast 20,000 under D + M treatment; (**G**,**H**) are chloroplast 5000 and 2000 under D + P treatment.

Compared to the control, drought stress induced noticeable changes in the ultrastructure of chloroplasts in sorghum leaves. Under normal conditions, the chloroplasts in the leaves of sorghum seedlings are oval-shaped, with clearly visible thylakoid and stroma lamellae closely aligned along the cell periphery, adjacent to the plasma membrane (Figure 7A,B). Drought stress caused the chloroplasts to swell, with the lamellar structures becoming loose and some disintegrating into indistinctness (Figure 7C,D). The application of exogenous melatonin under drought stress significantly mitigated the impact, with the chloroplasts slightly enlarged and the thylakoids and stroma lamellae remaining relatively clear (Figure 7E,F). In contrast to drought stress, the application of melatonin antagonist led to further disintegration and disappearance of some chloroplast lamellar structures, accompanied by an increase in volume (Figure 7G,H).

### 3.3. Effects of Exogenous Melatonin on DBA Staining, Membrane Damage and Antioxidant System in Leaves of Sorghum Seedlings under Drought Stress

#### 3.3.1. Effects of Exogenous Melatonin on DAB Staining of Sorghum Seedling Leaves under Drought Stress

**Figure 8:**
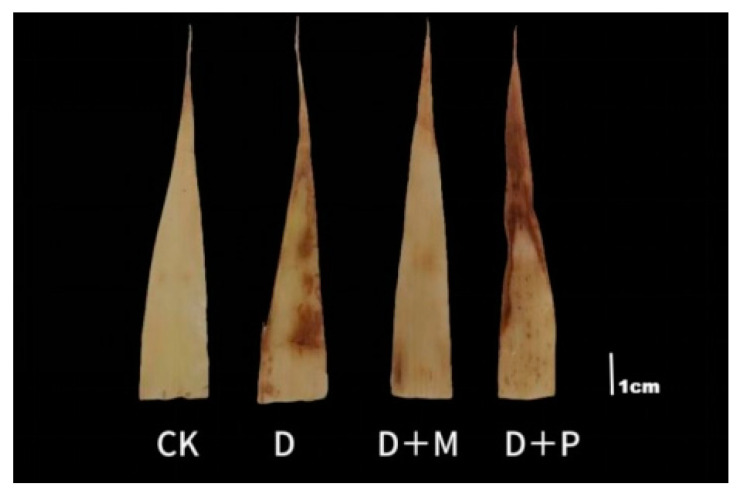
Effects of melatonin spraying on DAB staining of sorghum seedling leaves under drought stress. CK: control treatment; D: 20% PEG; D + M: drought + 100 μmol/L of MT; D + P: drought + 100 μmol/L of CPA.

Under normal conditions, the leaves of sorghum seedlings exhibit a light brown (Figure 8). In contrast to the control, the leaves of sorghum seedlings under drought stress displayed distinct yellow-brown spots, with the leaves appearing more yellow overall. After the application of exogenous melatonin under drought stress, the yellow-brown spots on the leaves were significantly reduced, and the overall leaf color was lighter. However, when a melatonin antagonist was applied under drought stress, the yellow-brown spots on the leaves merge, and the overall leaf color deepens. The DAB staining indicated that the application of exogenous melatonin endowed sorghum leaves with a stronger capacity to scavenge H_2_O_2_ and O_2_.

#### 3.3.2. Effects of Exogenous Melatonin on Leaf Cell Membrane Damage of Sorghum Seedlings under Drought Stress

**Figure 9:**
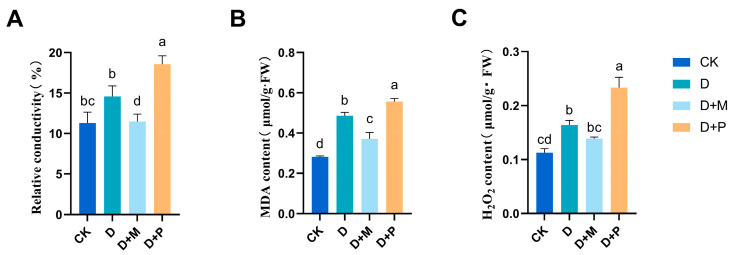
Effect of melatonin spraying on relative conductivity, MDA content, and hydrogen peroxide content in sorghum seedlings under drought stress: (**A**) relative conductivity; (**B**) MDA content (**C**) hydrogen peroxide content. CK: control treatment; D: 20% PEG; D + M: 20% PEG + 100 μmol/L of MT; D + P: 20% PEG + 100 μmol/L of CPA; columns represent mean ± SD, and, according to the LSD test, *n* = 3, significant differences are indicated in lower case letters (*p* ≤ 0.05).

Drought stress significantly increased the malondialdehyde (MDA) content and relative electrical conductivity in the leaves of sorghum seedlings (Figure 9). The MDA content and relative electrical conductivity increased significantly by 71.97% and 29.20%, respectively, under drought stress. The application of exogenous melatonin under drought conditions effectively mitigated these increases, leading to significant reductions of 23.66% in MDA content and 21.27% in relative electrical conductivity. Conversely, the application of melatonin antagonist under drought stress also increased the MDA content and relative electrical conductivity of the leaves, with significant increases of 14.68% and 27.25%, respectively, compared to the drought stress.

#### 3.3.3. Effects of Exogenous Melatonin on Antioxidant Enzyme Activity in Leaves of Sorghum Seedlings under Drought Stress

**Figure 10:**
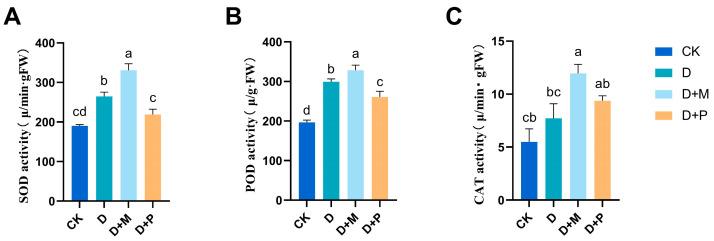
Under drought conditions, exogenous melatonin promoted the increased activity of SOD, POD and CAT in sorghum leaves: (**A**) SOD activity; (**B**) POD activity; (**C**) CAT activity. CK: control treatment; D: 20% PEG; D + M: 20% PEG + 100 μmol/L of MT; D + P: 20% PEG + 100 μmol/L of CPA; columns represent mean ± SD, and, according to the LSD test, *n* = 3, significant differences are indicated in lower case letters (*p* ≤ 0.05).

Drought stress notably influenced the activities of superoxide dismutase (SOD), peroxidase (POD), and catalase (CAT) in the leaves of sorghum seedlings (Figure 10). Compared to the control, the activities of SOD, POD, and CAT under drought stress all increased, with significant increases of 38.70%, 52.22%, and 40.64%, respectively. Compared to drought stress, the application of exogenous melatonin further augmented the activities of SOD, POD, and CAT in the leaves of sorghum seedlings, increasing by 25.06%, 9.76%, and 54.78%, respectively. Conversely, the application of melatonin antagonist under drought stress led to a significant suppression of these enzymatic activities of POD, SOD, and CAT, with significant decreases of 17.14%, 12.77%, and 21.19%, respectively, compared to drought stress.

#### 3.3.4. Effects of Exogenous Melatonin on Antioxidant System in Chloroplasts of Leaves of Sorghum Seedlings under Drought Stress

**Figure 11:**
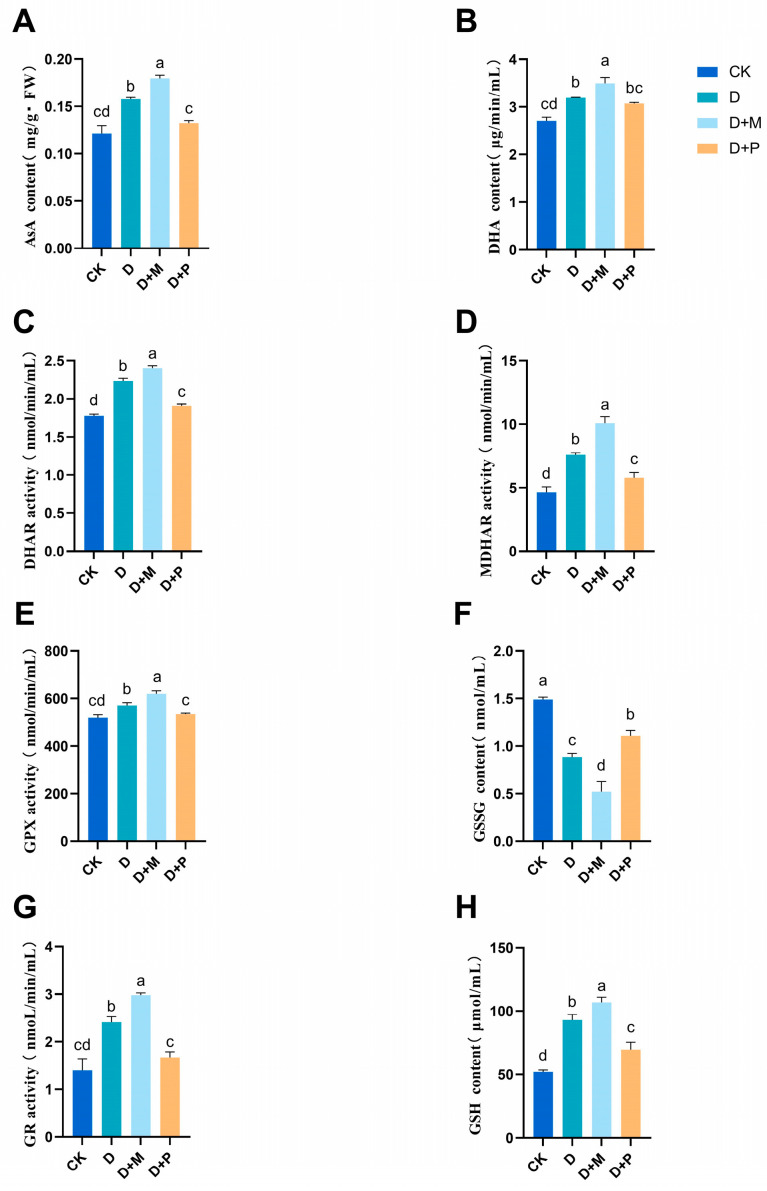
Under drought stress, melatonin affects the antioxidant content in sorghum chloroplast: (**A**) AsA content; (**B**) DHA content; (**C**): DHAR content; (**D**): MDHAR content; (**E**): GSH content; (**F**): GSSG content; (**G**) GPX content; (**H**) GR content.CK: control treatment; D: 20% PEG; D + M: 20% PEG + 100 μmol/L of MT; D + P: 20% PEG + 100 μmol/L of CPA; columns represent mean ± SD, and, according to the LSD test, *n* = 3, significant differences are indicated in lower case letters (*p* ≤ 0.05).

Drought stress significantly affected the activity of antioxidant enzyme and contents of non-enzymatic antioxidants within the chloroplasts (Figure 11). Compared with the control, the activities of DHAR, MDHAR, GPX, and GR in chloroplasts of sorghum leaves under drought stress were significantly increased by 25.84%, 63.65%, 10.13%, 72.49%, respectively. Similarly, the contents of AsA, DHA, GSH and, GSSH in chloroplasts of sorghum leaves under drought stress were significantly increased by 29.65%, 17.76%, 78.82%, and 78.82%, respectively. Under drought stress, the application of exogenous melatonin also significantly increased the activities of enzymatic antioxidants, among which the contents of MDHAR and GR were the most significantly increased by 32.84% and 23.55%, respectively. Under drought stress, the melatonin antagonist decreased the content of non-enzymatic antioxidants, and GSH contents significantly decreased by 25.05%. This indicated that the application of exogenous melatonin can influence the content of non-enzymatic antioxidant substances in chloroplasts, thereby enhancing the plant’s antioxidant capacity and improving its drought resistance.

### 3.4. Effects of Exogenous Melatonin on Contents of Proline and Soluble Sugar in Leaves of Sorghum Seedlings under Drought Stress

**Figure 12:**
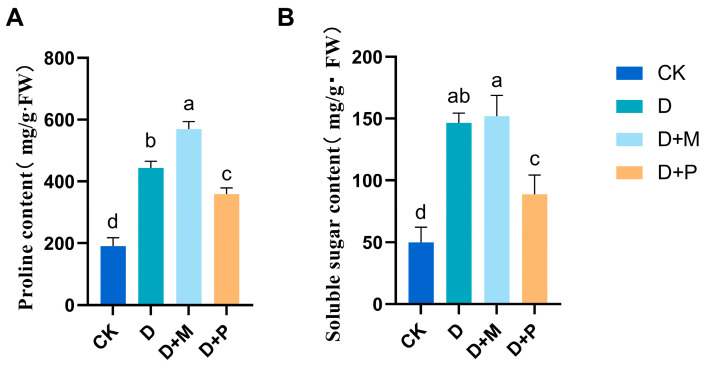
Under drought conditions, exogenous melatonin promoted the increase in proline and soluble sugar content in sorghum leaves: (**A**) free proline content; (**B**) soluble sugar content. CK: control treatment; D: 20% PEG; D + M: 20% PEG + 100 μmol/L of MT; D + P: 20% PEG + 100 μmol/L of CPA; columns represent mean ± SD, and, according to the LSD test, *n* = 3, significant differences are indicated in lower case letters (*p* ≤ 0.05).

Under drought stress, there was a significant increase in the content of proline and soluble sugars in sorghum seedling leaves (Figure 12). Compared to the control, the content of proline and soluble sugars in the leaves of sorghum seedlings under drought stress significantly increased by 132.96% and 194.87%, respectively. In comparison to drought stress, the application of exogenous melatonin further significantly increased the proline content in the leaves of sorghum seedlings by 28.33%. Conversely, the application of melatonin antagonist under drought stress led to a significant decrease in both proline and soluble sugar content in the leaves of sorghum seedlings by 19.14% and 39.40%, respectively.

### 3.5. Effects of Exogenous Melatonin on Melatonin Content of Sorghum Seedling Leaves under Drought Stress

**Figure 13:**
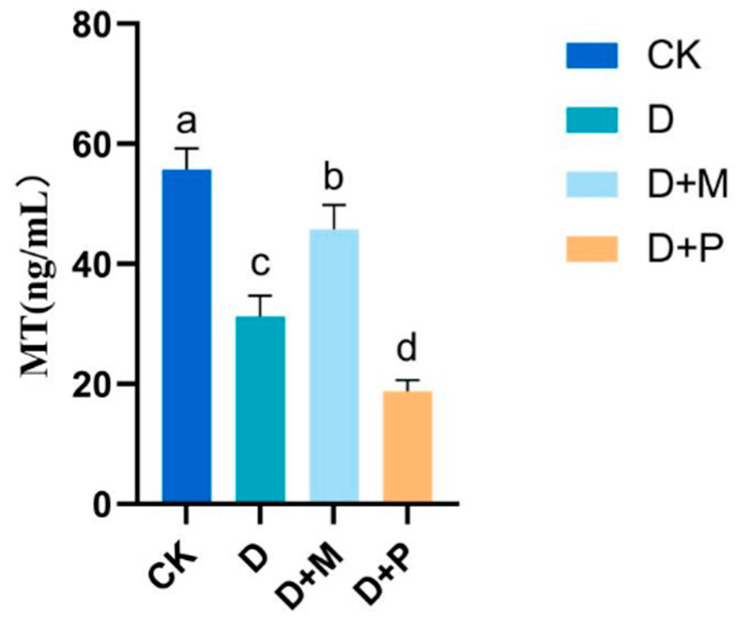
Under drought conditions, exogenous melatonin promoted the increase in melatonin content in sorghum leaves. CK: control treatment; D: 20% PEG; D + M: 20% PEG + 100 μmol/L of MT; D + P: 20% PEG + 100 μmol/L of CPA; columns represent mean ± SD, and, according to the LSD test, *n* = 3, significant differences are indicated in lower case letters (*p* ≤ 0.05).

Drought stress significantly reduced the melatonin content in the leaves of sorghum seedlings (Figure 13). Compared to the control, the melatonin content in drought-stressed sorghum seedlings significantly decreased by 43.99%. The application of exogenous melatonin under drought stress increased the melatonin content in the leaves of sorghum seedlings, with a significant increase of 43.67%. Conversely, the application of melatonin antagonist under drought stress also reduced the melatonin content in the sorghum seedlings, causing a decrease of 39.79% in melatonin content in the leaves.

### 3.6. Exploring the Correlation between the Treatment and the Measurement Indexes by Cluster Heat Maps and Principal Component Analysis

**Figure 14:**
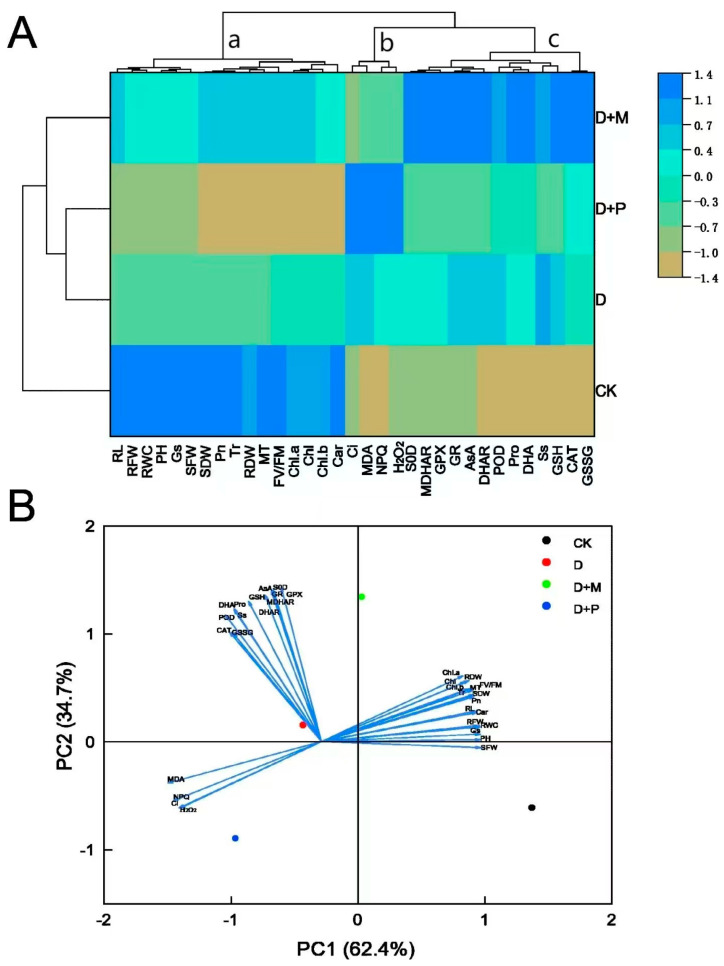
(**A**) The cluster heatmap shows a comprehensive visual overview of the responses of different parameters to different treatments, using the normalized mean values to facilitate effective comparisons. (**B**) Principal component analysis (PCA) analyzes the complex relationship between processing and parameters. Figure (**B**), the two components of PC1 and PC2 together accounted for 97.10% of the variance in the data set. Positive or negative correlations between various morphological physiological and biochemical parameters and various treatments are illustrated by the vector lines of the biplot. Small angles indicate a weak association, while large angles indicate a strong association. The parameters include PH (plant height), RL (root length), SFW (shoot fresh weight), RFW (root fresh weight), SDW (shoot dry weight), RDW (root dry weight), RWC (leaf relative water content), Pn (photosynthetic rate), Tr (transpiration rate), Ci (intercellular carbon dioxide concentration), Gs (stomatal conductance), Chl (total chlorophyll), Chl. a (chlorophyll a), Chl.b (chlorophyll b), Car (carotenoids), Fv/Fm, NPQ, RC (relative conductivity), SOD, POD, CAT, MDA, GR, GSSG, GSH, GPX, AsA, DHA, DHAR, MDHAR, MT (melatonin), Pro (proline), and Ss (soluble sugars).

The performance of various parameters under different treatment conditions was exhibited by a heat map, and then the parameters were divided into three different clusters using a hierarchical clustering method (Figure 14A). The distinct responses of the parameters within each cluster to the drought stress (D) and the subsequent effects of exogenous melatonin application (D + M) and the application of the melatonin antagonist (D + P) have been specified. For instance, parameters within cluster a, which are primarily associated with plant growth and water status, showed a decline under drought stress. However, the cluster-a parameters showed an upward trend after melatonin spraying under drought stress, while the cluster-a parameters showed a downward trend after the application of melatonin antagonist, highlighting the importance of endogenous melatonin in stress mitigation. The cluster-b parameters, primarily indicative of cellular damage, showed an increasing trend under drought stress treatment. Nevertheless, the cluster-b parameters showed a decreasing trend after melatonin spraying, while the cluster-b parameters showed an increasing trend after application of melatonin antagonist. The cluster-c parameters, mainly related to antioxidant activity, showed an increasing trend under drought stress treatment. However, the cluster-c parameters showed an upward trend after melatonin spraying, while the cluster-c parameters showed a downward trend after the application of melatonin antagonist. Thereafter, to examine the relationship between different treatments and variables, principal component analysis was carried out. PC1 (61.9%) and PC2 (35.2%) together accounted for a large portion of the variability, accounting for 97.1% of the total variability observed in the data. The significant effects of the experimental treatments on sorghum plant growth were clearly distributed along the PC1 axis in the order of CK > D + M > D > D + P, implying that exogenous melatonin treatment could significantly alleviate drought stress. Notably, the cluster-c parameters had higher PC2 scores and showed a substantial correlation with the D + M treatment. However, the cluster-b parameters had lower scores in both PC1and PC2, and showed a close correlation with the D + P treatment. Interestingly, the cluster-a parameters indicated a strong affinity with the CK.

## 4. Discussion

Under drought stress, the morphological and physiological–biochemical characteristics of crops will change to adapt to the stressful environment [43,44]. In this experiment, drought stress significantly inhibited the growth of sorghum seedlings, while the application of exogenous melatonin significantly improved the growth condition of sorghum seedlings under drought stress. Specifically, after melatonin treatment, the plant height and root length, as well as the fresh and dry weight of the shoots and roots of the sorghum seedlings, all significantly increased (Figure 1). This indicated that exogenous melatonin may enhance the adaptability to drought by regulating the balance of plant root-to-shoot development [45,46]. The growth of sorghum seedlings was inhibited when melatonin antagonists were sprayed under drought stress, further proving that melatonin plays a key role in promoting the growth of sorghum seedlings under drought stress.

Drought stress often disrupts the osmotic balance within plant cells [47,48], and the ability to promptly eliminate excess reactive oxygen species (ROS) is crucial for maintaining the normal physiological functions and growth of sorghum. Oxidative stress can damage cellular structures and induce the generation of ROS, and an excess of ROS can cause irreversible damage to chloroplasts and membrane lipids, thereby harming plant cells [49,50]. In this experiment, under drought stress, there was a significant increase in the relative conductivity and malondialdehyde (MDA) content of sorghum seedling leaves, indicating that the cell membrane structure was damaged. The DBA staining also reflected the effects of oxidative stress. Concurrently, the activities of superoxide dismutase (SOD), peroxidase (POD), and catalase (CAT) in the leaves of sorghum seedlings showed an upward trend after drought stress, indicating that drought stress can stimulate the activity of antioxidant enzymes in sorghum to clear accumulated ROS. After the application of melatonin, the activity of these antioxidant enzymes was even higher, suggesting that melatonin endowed sorghum seedlings with a stronger capacity to clear ROS and reduce damage to the cell membrane. Moreover, principal component analysis indicated a significant positive correlation between the treatment of drought-stressed sorghum seedlings with exogenous melatonin and the activity and/or levels of enzymatic and non-enzymatic antioxidants.

Chloroplasts are the primary source of ROS, and damage to their structure is mainly caused by ROS [51]. After drought stress, the chloroplasts in the leaves of sorghum seedlings became deformed and swollen, with the disintegration of the double membrane structure and a blurring of the thylakoid granum matrix lamellar structure. When the thylakoid stacks decrease or even disappear, it reduces the capacity for light energy absorption, transmission, and utilization, preventing light energy from being fully converted into chemical energy, thus reducing photosynthetic capacity [52]. However, after the application of melatonin under drought stress, the chloroplasts in the leaves of sorghum seedlings can still maintain an elongated oval shape, the matrix lamellae can still maintain a good arrangement, and the double membrane structure was clearly visible. This was mainly due to the increased activity of antioxidant enzymes after melatonin application, simultaneously the increased content of non-enzymatic antioxidants in the chloroplasts, such as ascorbic acid and glutathione, which minimize the damage to chloroplasts by ROS. Changes in the morphology and structure of chloroplasts are an important factor affecting the chlorophyll content in sorghum [53]. Since chlorophyll is the main pigment for photosynthesis, its content also has a significant impact on the plant’s photosynthetic process [8,54]. In this experiment, drought stress significantly reduced the chlorophyll content in the leaves of sorghum seedlings, and the application of melatonin increased the chlorophyll content in the leaves of sorghum seedlings under drought stress, which was beneficial for the absorption and transmission of photosynthesis. At the same time, the application of melatonin increased the photosynthetic rate under drought stress and reduced the intercellular CO_2_ concentration, indicating that melatonin enhanced the assimilation capacity for CO_2_.

Photosystem II (PSII) is considered particularly sensitive to environmental stress [55,56,57]. Studies have found that drought stress can damage the reaction center of Photosystem II, reducing the efficiency of primary light energy conversion (Fv/Fm). A decrease in Fv/Fm also indicates that the reaction center of Photosystem II has suffered some degree of damage [58,59]. In this experiment, drought stress led to a declining trend in sorghum’s Fv/Fm, implying a compromised light energy conversion efficiency. This was attributed to damage to PSII, inhibition of electron transport, and a consequent decrease in photosynthetic activity. However, the application of exogenous melatonin increased the Fv/Fm in sorghum and decreased the non-photochemical quenching (NPQ), indicating that the Photosystem II was repaired and photosynthetic activity was enhanced. NPQ serves as an indicator of the extent to which light energy is transformed into chemical energy. A lower NPQ signified a higher rate of re-oxidation of primary electron acceptors from QA- to QA, which in turn reflected a mitigation of the adverse effects of drought stress on the electron transport activity of sorghum’s PSII following melatonin application. Furthermore, the PCA revealed a significant negative correlation between the application of exogenous melatonin to sorghum seedlings and NPQ. This correlation was mirrored in the observed alterations in the electron transport rate and the characteristics of the OJIP curve, providing further evidence of the protective role of melatonin in enhancing the resilience of sorghum to drought-induced stress.

The water status within crops significantly influences their physiological and biochemical processes. Zhang et al. (2019) [54] showed that under water stress, there is a significant correlation between the RWC of sorghum leaves and the net photosynthetic rate. A reduction in the relative water content of leaves caused by drought can decrease the net photosynthetic rate [60]. In this study, the application of exogenous melatonin significantly increased the RWC of sorghum leaves under drought stress. Additionally, the application of melatonin enhanced the content of osmotic regulatory substances, which helped to reduce water loss and thus maintained normal physiological metabolism. Cui et al. (2022) [61] showed that *Dalbergia odorifera* may increase soluble sugar production through enhanced starch degradation, using these sugars as compatible solutes for osmotic adjustment. PCA also clearly showed that RWC had a positive effect on sorghum plant growth, and was positively correlated with physiological indexes to maintain normal plant growth under drought stress.

## Data Availability

Data is contained within the article.
